# Sex Differences in Intestinal Microbial Composition and Function of Hainan Special Wild Boar

**DOI:** 10.3390/ani10091553

**Published:** 2020-09-02

**Authors:** Xiaozhe Wang, Ying Zhang, Qiong Wen, Ying Wang, Zhixin Wang, Zhen Tan, Kebang Wu

**Affiliations:** 1 College of Animal Science and Technology, Hainan University, Haikou 570228, China; 18090501210004@hainanu.edu.cn (X.W.); zy18208949823@163.com (Y.Z.); qiong1590399@163.com (Q.W.); wy_1428@163.com (Y.W.); wzx13856583860@163.com (Z.W.); 2 Laboratory of Tropical Animal Breeding, Reproduction and Nutrition, Hainan University, Haikou 570228, China

**Keywords:** special wild boar, intestinal microorganisms, 16S rRNA gene, sex differences, castration

## Abstract

**Simple Summary:**

The gut microbiome plays an important role in the health and disease status of the host. This study explores the effect of sex on intestinal microorganisms of Hainan special wild boar, hybrids of wild boar, and local sows in Hainan. Results showed that there were sex differences in the composition and function of intestinal microorganisms. Our research provides a foundational reference for the future research on Hainan special wild boars and on sex differences in the microbiomes of other species.

**Abstract:**

The gut microbiome plays an important role in the health and disease status of the host. Research on the effect of sex on animal intestinal microorganisms is still limited; and the effect of castration on the gut microbiome of male pigs has not been fully investigated. In this study, 30 Hainan special wild boars at the same growth stage were divided into three groups (10 entire males, 10 females, and 10 castrated males). High-throughput 16S rRNA sequencing was used to investigate the fecal microbiota of the Hainan special wild boar. Firmicutes, Bacteroidetes, Actinobacteria, Spirochaetes, and Proteobacteria were the five dominant phyla found in the specimens. The relative abundance of Bacteroidetes was higher in the microbiota of female pigs than in male pigs, while Firmicutes was on the contrary. The percentage of *Streptococcus* and *Lactobacillus* was higher in males than females. The microbial diversity of females was significantly higher compared to males; castration increased the intestinal microbial diversity of males. Functional prediction showed that male fecal microorganisms were rich in membrane transport and carbohydrate metabolism; energy metabolism, glycan biosynthesis, and metabolism of cofactors and vitamins were rich in the female group; the fecal microorganisms of castrated males had higher membrane transport abundance.

## 1. Introduction

The animal gut microbiome is regulated by many factors, including environmental variables and host genetics [1,2,3]. Although sex may be one of these important factors, research on the impact of sex on the intestinal microorganisms of animals has produced varying results. Some studies have shown that sex has little or no effect on intestinal microorganisms [4,5]. For example, a study in mouse revealed that genetic background has a significant impact on the microbial composition and exerts a stronger influence than sex [4]. Other studies, however, have clearly shown sex-specific differences in the composition of the gut microbiome [6,7,8,9,10,11]. A recent study showed that the relative abundance of Actinobacteria and Tenericutes in male mice was higher than that in female mice. At the genus level, *SMB53* from family Colstridiaceae and three members of family Lachnospiraceae (*Dorea*, *Coprococcus*, and *Ruminococcus*) were found to be more abundant in females than in males [8]. One study on mammals found that sex affects the composition of the intestinal microbial community [12], and another study showed that different sexes have different community composition mainly due to the differences in *Bacteroides* and *Prevotella* abundance [13]. Several studies have also suggested sex can regulate the composition of intestinal microorganisms through hormone-microbe interactions and sex-specific immune responses [14], and further research revealed that sex hormones can directly affect the composition of intestinal flora [15]. Similarly, researchers have previously stated that castration changes the composition of gut microbial community due to a deficiency in androgen [16]. Collectively, these studies suggest that sex plays an important role in shaping the gut microbiome in animals.

The Hainan special wild boar results from crossing of a male (♂) Hainan wild boar (wild boar in Hainan Province of China) and a female (♀) Tunchang pig (a local breed of domestic pig in Hainan Province of China) to produce an F1 generation. Female individuals are then selected from the F1 generation and are bred with male Hainan wild boars to carry out secondary hybridization. The hybrid F3 generation is used for subsequent commercial breeding, producing subsequent generations that carry 7/8 wild boar ancestry and 1/8 domestic pig ancestry. Previous laboratory members’ studies on Hainan special wild boars showed that males grew faster than females at the same growth stage, and their body weight was significantly higher than that of females. As an endemic pig in Hainan Province, the Hainan special wild boar is prized for its tender and rich meat. In addition, the Hainan special wild boar has a high economic value, which is highly significant to the development of the local economy.

To our knowledge, there is no research on the gut microbiome of the Hainan special wild boar. In this study, we explore sex differences in the composition and function of intestinal microorganisms in the Hainan wild boar using 16S rRNA gene sequencing. Our findings provide an important reference for more in-depth studies on the complex interactions between host and microbiome in non-model species.

## 2. Materials and Methods

### 2.1. Experimental Animals and Sample Collection

The animals in this study all came from the Yulvbao Wild Boar Breeding Center in Changjiang Li Autonomous County, Hainan Province, China. The study was conducted in strict accordance with the Guidelines for Experimental Animals issued by the Ministry of Science and Technology (Beijing, China), and all animal experiments were approved by the Institutional Animal Care and Use Committee of Hainan University. All methods strictly follow the regulations of the People’s Republic of China for quality Supervision, Inspection and Quarantine (GB/T 17236-2008). All experimental pigs were 8 months old and weighed about 45–55 kg. The boar was castrated at the age of 7 days, and the castration was done surgically. Before fattening, pigs were fed with similar artificial control conditions and the same commercial formula feed. During the fattening stage, all pigs were raised in the same fattening room and allotted in pen contained 4–5 pigs/pen. The fattening room was semi-enclosed, and the ambient temperature was between 15–25 °C. Pigs received the same diets specific for fattening pigs (Supplementary Table S1), diets were administered twice a day. Each pen was equipped with nipple drinkers with ad libitum access to fresh water. All the animals were healthy and did not receive any antibiotics.

A total of 30 samples were collected from the feces of entire (uncastrated) male pigs (EM, n = 10), female pigs (FE, n = 10) and castrated male pigs (CM, n = 10). Fresh fecal samples were collected from all individual pigs by professionals within three days. Each fresh fecal sample was stored in a 2 mL centrifuge tube and immediately flash frozen in liquid nitrogen. After collection, all samples were stored in a −80 °C freezer for cryopreservation until DNA extraction.

### 2.2. DNA Extraction and PCR Amplification

The QIAamp DNA Stool Mini Kit (Qiagen Ltd., Hilden, Germany) was used to extract DNA from fecal samples, following the standard protocol provided by the kit manufacturer. The V3 + V4 region of the bacterial 16S rRNA gene was amplified by PCR with forward primer 338F (5′-ACTCCTACGGGAGGCAGCA-3′) and reverse primer 806R (5′-GGACTACHVGGGTWTCTAAT-3′). The following 10 μL reaction was used for PCR: 50 ng genomic DNA, 0.3 μL forward primer (10 μM), 0.3 μL reverse primer (10 μM), 5 μL KOD FX Neo Buffer, 2 μL dNTP (2 mM), 0.2 μL KOD FX Neo. The thermocycling protocol was as follows: 95 °C for 5 min, 20 cycles of 95 °C for 30 s, 50 °C for 30 s, and 72 °C for 40 s, with a final extension at 72 °C for 7 min. The 2% agarose gel electrophoresis was used to confirmed that the PCR products had good quality and concentration. Then, samples with the main band brightness of 400–440 bp were selected for further experiments. The constructed library was sequenced on an Illumina HiSeq 2500.

### 2.3. 16S rRNA Gene Sequencing and Sequence Splicing

Sequencing was carried out by Beijing Biomarker Technologies. The original image data file obtained by high-throughput sequencing was converted into sequences by base recognition analysis and stored in FASTQ file format. FLASH version 1.2.7 [17] was used to assemble the sequence of each sample, then the obtained sequence was spliced by Trimmomatic version 0.33 [18], and finally the chimera sequence was identified and removed by UCHIME version 4.2 [19].

### 2.4. Species Annotation and Taxonomic Analysis

Using USEARCH version 10.0 [20], we clustered sequences by at least 97% similarity to obtain operational taxonomic units (OTUs). The species classification information corresponding to each OTU was obtained by comparing the representative sequence of OTU with the microbial reference database. Taxonomic annotation of the OTUs was done based on the bacterial taxonomic database provided by Silva [21]. We filtered out OTUs that were less than 0.005% of total sequences [22] to create the final OTU list. With this, we assessed the relative abundance of each OTU in each sample by counting the number of tags from each OTU. Community composition for each sample was then assessed at the phylus and genus level. A species abundance table was generated with QIIME version 1.8.0 [23] and the corresponding community structure map was drawn in R.

### 2.5. Diversity Analysis and Significant Difference Analysis between Groups

Mothur version v.1.30 [24] was used to evaluate the alpha diversity index of samples. Beta diversity analysis was conducted with QIIME by comparing the similarity of species diversity among different samples. Unweighted Pair-group Method with Arithmetic Mean (UPGMA) was used in R to cluster the samples, allowing us to judge the similarity of species composition among samples. Non-metric Multi-Dimensional Scaling (NMDS) [25] was used to show the species diversity differences among samples. Line Discriminant Analysis Effect Size (LEfSe) [26] was used to find species with significant differences in abundance among different groups. Analysis of Variance (ANOVA) was adapted to significant differences in mean among groups.

### 2.6. Predictive Analysis of Functional Genes

The Kyoto Encyclopedia of Genes and Genomes (KEGG) and Clusters of Orthologous Groups of proteins (COG) databases were used to predict the functions of all the OTUs. By comparing the species composition information obtained from sequencing data by using PICRUSt [27]. We can infer the composition of functional genes in the samples, so as to analyze the functional differences among different samples or groups [28]. The abundance of KEGG and COG were then calculated, followed by calculations of the abundance of each functional category. At the genus level, a *t*-test test was performed between different groups, and the *p*-value significance threshold was set to 0.05.

## 3. Results

### 3.1. OTU Clustering and Species Taxonomy Analysis

**Figure 1:**
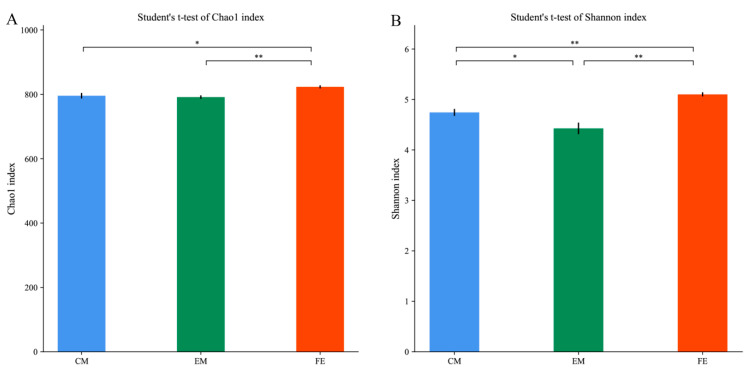
Dynamic changes in intestinal microorganism alpha diversity. (**A**) Chao1 index; (**B**) Shannon index. Different letters represent significant differences in alpha diversity indices based on Student’s *t*-test (* *p* < 0.05, ** *p* < 0.01).

After quality filtering, we obtained 5,656,950 sequences from 30 fecal samples and each sample contained 188,565 sequences on average (Supplementary Table S2). A total of 859 OTUs were detected, of which 856 were shared among all three groups (i.e., female, castrated male, entire male). According to our rarefaction curve, our sequencing depth was sufficient across samples to detect the OTUs that were present (Supplementary Figure S1). The Chao1 index and Shannon index, measures of alpha diversity, were highest in female pigs. The Shannon index was lowest in entire male pigs (Figure 1).

**Figure 2:**
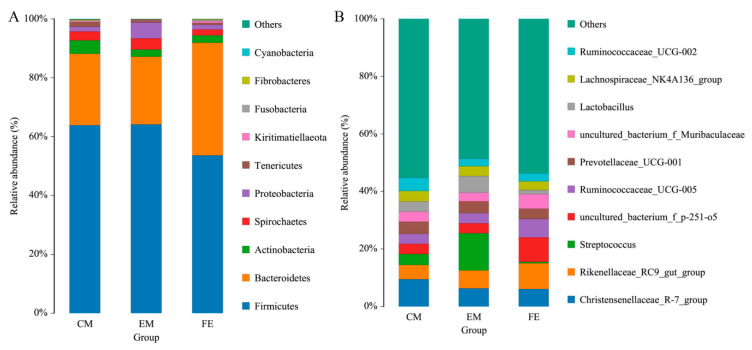
Histogram of the top 10 phylum (**A**) and genus (**B**) in each group.

Taxonomic analysis showed that Firmicutes, Bacteroidetes, Actinobacteria, Spirochaetes, and Proteobacteria were the most abundant intestinal microorganisms in all three groups. The proportion of these five phyla was 98.80% in entire male pigs, 98.00% in female pigs and 97.38% in castrated male pigs. Of these, Firmicutes and Bacteroidetes constituted the two dominant phyla of intestinal microorganisms. The proportion of Firmicutes in entire male pigs (64.17%) and castrated male pigs (63.89%) was significantly higher than in female pigs (53.65%). On the other hand, the proportion of Bacteroidetes in female pigs (38.22%) was higher than in entire male pigs (22.96%) and castrated male pigs (24.26%) (Figure 2A). Compared with castrated males (4.55%) and females (2.55%), the proportion of Actinobacteria was higher in entire males (2.47%) (2.47% in EM, 4.55% in CM and 2.55% in FE). In addition, the relative abundance of Proteobacteria and Spirochaetes in entire male pigs was the highest among the three groups, followed by castrated male pigs.

At the genus level, the relative microfloral abundances differed across the three groups of pigs (Figure 2B). For example, the proportion of *Christensenellaceae_R-7_group* was 6.34% in entire male pigs, 6.10% in female pigs and 9.52% in castrated male pigs. The proportion of *Streptococcus* in entire male pigs (12.93%) was significantly higher than in female pigs (0.51%) and castrated male pigs (3.84%). The proportion of *Rikenellaceae_RC9_gut_group* in females (8.93%) was higher than in entire males (6.20%) and castrated males (4.92% in CM). The proportion of *uncultured_bacterium_f_p-251-o5* in female pigs was 8.49%, higher than in entire male pigs (3.47%) and castrated male pigs (3.52% in CM). The proportion of *Lactobacillus* in was 5.76% in entire male pigs, 1.45% in female pigs and 3.55% in castrated male pigs. Most species belonged to Firmicutes, followed by Bacteroidetes, Proteobacteria, Actinobacteria (Supplementary Figure S2).

### 3.2. Comparison of Fecal Microorganisms among Three Groups of Pigs

**Figure 3:**
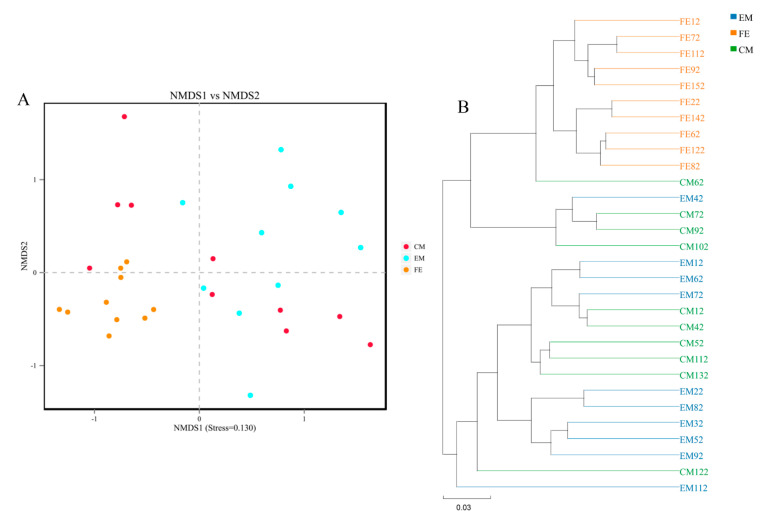
NMDS ordination (**A**) and UPGMA cluster (**B**).

NMDS revealed differences in microbial profiles based on Weighted Unifrac dissimilarity (Figure 3A). Despite the lack of uniformity of the distribution, the results still showed some differences. The microbiomes of entire male pigs and female pigs gathered in separate groups. Some of the samples in the castrated male pigs were clustered with the entire male pig samples, while others were closer to the female pig samples. Based on the Weighted Unifrac dissimilarity, UPGMA showed that the microbiomes of female pigs gathered in one cluster, while those in entire male pigs (except sample EM42) gathered in another cluster (Figure 3B). Most of the microbiomes of castrated male pigs were clustered with those of entire male pigs, although a few were clustered with female pigs.

**Figure 4:**
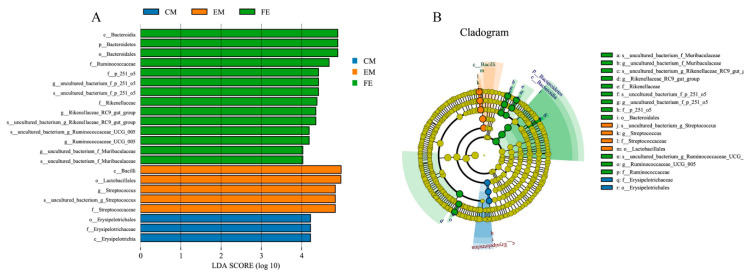
LDA value distribution histogram (**A**) and LEfSe analysis evolution branch diagram (**B**).

LEfSe can find biomarkers with statistical differences among different groups [25]. Figure 4A is a histogram of Line Discriminant Analysis (LDA) value distribution, showing species with LDA Score greater than 4.0. A total of 22 biomarkers with statistical differences were detected by LEfSe (5 in EM, 14 in FE, and 3 in CM). The significant biomarkers in entire male pigs and castrated male pigs were all distributed in Firmicutes, while the 14 significant biomarkers in female pigs were mainly distributed in Bacteroidetes and Firmicutes. The LEfSe cladogram analysis revealed that 18 biomarkers of different classification levels were significantly different among the three groups (Figure 4B). ANOVA showed that there were significant differences in 3 phyla between entire males and females, 1 phylum between entire males and castrated males, and 2 phyla between females and castrated males (Supplementary Figure S3). Among the 20 genera with the lowest *p*-value, 18 genera had significant differences between entire males and females, 3 genera had differences between entire males and castrated males, and 17 genera had differences between females and castrated males (Supplementary Figure S4).

### 3.3. Differences of Microbial Function among Three Groups of Pigs

**Figure 5:**
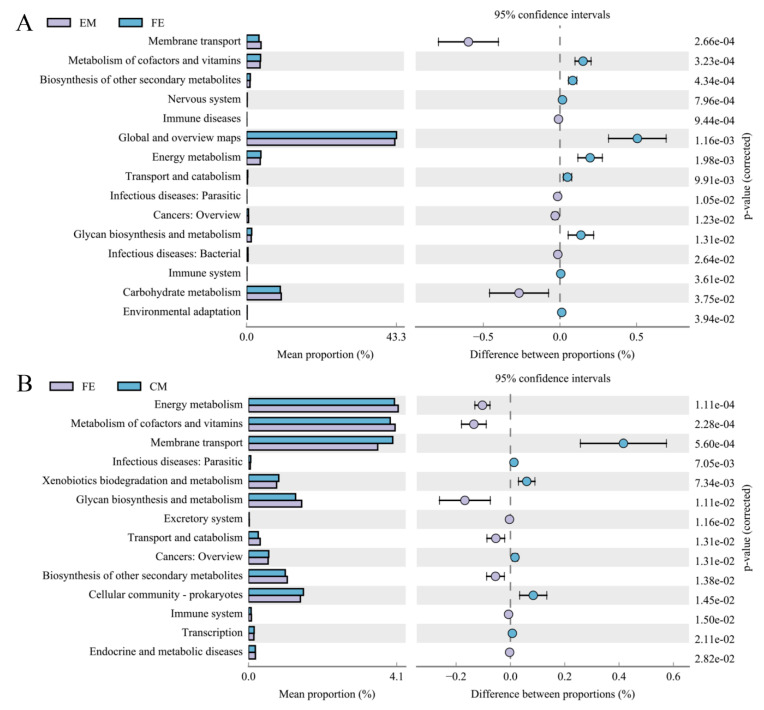
Analysis of the difference of KEGG metabolic pathway between groups at the second level. (**A**) comparison between entire male pigs and female pigs; (**B**) comparison between female pigs and castrated male pigs. Different colors represent different groups. The left figure in the picture shows the abundance ratio of different functions in two groups of samples, the middle shows the difference ratio of functional abundance in the 95% confidence interval, and the right value is the *p*-value.

By analyzing the differences of KEGG metabolic pathways, we can detect differences in metabolic pathways of functional microbial genes between different groups of samples. A total of 46 KEGG metabolic pathways were analyzed. The results showed that there was no significant difference in microbial community function between entire male pigs and castrated male pigs. However, there were significant differences in 15 microbial metabolic pathways between entire male pigs and female pigs (Figure 5A). Membrane transport, metabolism of cofactors and vitamins, biosynthesis of other secondary metabolites, nervous system, and immune diseases were the five most significant metabolic pathways. The abundance of 6 functions in entire male pigs was higher than in females, including membrane transport and carbohydrate metabolism. The abundance of the other 9 functions was higher in female pigs. In addition, a total of 14 microbial metabolic pathways were different between female pigs and castrated male pigs (Figure 5B). Energy metabolism, metabolism of cofactors and vitamins, and membrane transport were the three most significant differences. There were 8 functions with higher abundance in female pigs and 6 functions with higher abundance in castrated male pigs.

**Figure 6:**
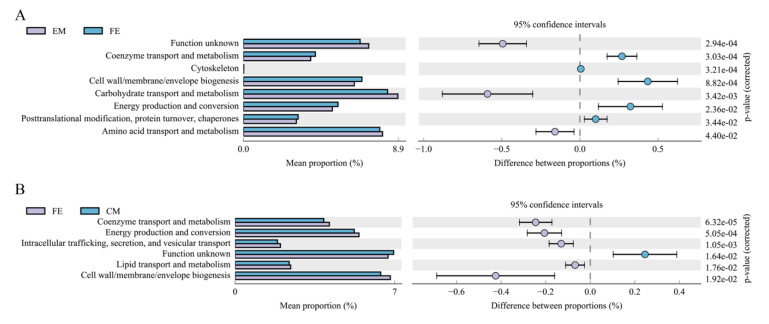
COG function analysis diagram. (**A**) comparison between entire male pigs and female pigs; (**B**) comparison between female pigs and castrated male pigs.

COG is a common protein functional classification database for prokaryotes. Using this, a total of 25 metabolic pathways was studied. No significant difference was found between entire male pigs and castrated male pigs. There were significant differences in 8 pathways between entire male pigs and female pigs (Figure 6A). Cell wall/membrane/envelope biogenesis, energy production, and conversion and other three pathways were more abundant in female pigs. There were significant differences in 6 metabolic pathways between female pigs and castrated male pigs (Figure 6B), with the abundance of five metabolic pathways higher in female pigs than in castrated male pigs.

## 4. Discussion

Our study reveals sex differences in the composition and function of intestinal microorganisms of the Hainan special wild boar. It is well known that sex differences have significant effects on the physiology and behavior of animals [29], but it has been more difficult to show that sex differences lead to differences in the gut microbiome of animals. Previous studies found that *Veillonellaceae, Roseburia*, *Bulleidia*, and *Escherichia* had higher abundances in boars, yet *Treponema* and *Bacteroides* were over-represented in gilts. Castration significantly shifted the fecal microbiota composition of the boars toward that of gilts [30]. Here, we carefully controlled the diet and environment of the Hainan special wild boars in our study, and were thus able to show significant differences in gut microbiome composition relating to sex.

Previous studies showed that there was no significant difference in intestinal microorganisms between sexes in the first few weeks of life [31], but that significant differences can arise after puberty [6]. Consistent with a previous study on mice, we found that females have higher microbial diversity than males [10]. Interestingly, we found that the microbial diversity of castrated males was higher than that of males and lower than that of females, and this difference was statistically significant.

Similar to the composition and structure of intestinal microbial communities in human and other mammals [32,33,34], Firmicutes and Bacteroidetes were the two dominant phyla found in the Hainan special wild boar gut microbiome. Notably, the relative abundance of Proteobacteria was higher in entire male pigs, and the relative abundance of Actinobacteria was higher in castrated male pigs. Previous studies showed that Proteobacteria is the dominant phylum in the gut microbiome of pandas and plays a key role in the digestion of lignin food sources [35,36]. A further study also pointed out that the abundance of Proteobacteria is very important for intestinal health [37]. Actinobacteria is associated with the synthesis of antibiotics, immunomodulatory compounds and metabolites, which are essential for animal health [38].

A study in pigs found that *Christensenellaceae_R-7_group* and *Rikenellaceae_RC9_gut_group* help digest soluble dietary fiber [39]. The proportions of *Christensenellaceae_R-7_group* and *Rikenellaceae_RC9_gut_group* in the Hainan special wild boar were high, which may be related to the genetic characteristics of crude fiber intake in Hainan wild boar. In addition, *Christensenellaceae_R-7_group* can promote intestinal development and barrier function, thus improving the growth performance of pigs [40]. *Streptococcus* plays an important role in the health of skin and mucus membranes of animals, and previous studies have shown that *Streptococcus* can improve immune function [41]. The abundance of *Streptococcus* in entire male pigs was higher, which may be the reason the functional abundance of immune diseases in males is higher than that in females. *Lactobacillus* is closely linked with bile acid metabolism and plays an important role in the absorption of dietary fat and vitamins [42]. In addition, it is reported that *Christensenellaceae_R-7_group* and *Ruminococcaceae_UCG-005* are related to the production of short-chain fatty acids [40,43]. *Christensenellaceae_R-7_group* can produce butyric acid, and butyric acid contributes to the relative mRNA expression and secretion of Mucin 2 [40]. *Ruminococcaceae_UCG-005* is thought to be a producer of acetic acid and butyric acid, and helps regulate hormone levels and inflammation [43]. Although the animals were normally healthy, environmental stimuli, such as nightlight, or the potential for disease, can also cause a stress response or differences in their hormone levels that might affect the structure of the gut microbiota, which were not monitored in this study.

NMDS analysis showed that there were some differences in the composition of intestinal microbiomes among the three groups. Intestinal microorganisms are affected by host hormones [16], and the secretion of androgens in animals decreases after castration. A previous study showed that the microflora of females and castrated males are closer to each other [6]. In our study, UPGMA analysis showed that some castrated male pig samples were clustered with female pig samples, while other castrated male pig samples were clustered with male pig samples. One possibility is that castrated males have varied levels of sex hormone secretion, though this needs to be investigated with further research.

Most potential biomarkers in entire male pigs and castrated male pigs were distributed in Firmicutes, while the biomarkers of female pigs were mainly distributed in Bacteroidetes. This may be related to the relatively high abundance of Bacteroidetes in female pigs. High abundance of Firmicutes and Bacteroidetes can promote the host’s food digestion process and contribute to energy intake [44,45,46,47,48]. Studies have shown that the intestinal microflora of entire male pig has a greater ability to use carbohydrates and proteins, while the intestinal microflora of female pigs promotes energy collection [30]. At some lower classification levels, there were significant differences in the composition of intestinal microbiomes between the sexes [49]. These differences in microbial species may lead to the observed differences in intestinal microbial diversity and functions of different groups.

Intestinal microbes contain about 600,000 genes, many more than those in the host genome. Therefore, intestinal microorganisms are considered to be an important organ of the host, directly contributing to health and participating in the construction of intestinal microecosystem [50]. This microecosystem performs different functions when the composition of intestinal microbial community changes. To analyze functional differences among different groups, we used the community species composition information to speculate on functional gene composition. The functional difference between the gut microbiomes of entire male pigs and castrated male pigs was not significant. We propose that this may be due to the similarity of their intestinal microbial composition.

Through the comparison of KEGG pathways, compared with males, the fecal microorganisms of females have functional enrichment in immune system, transport and catabolism, biosynthesis of other secondary metabolites, energy metabolism, metabolism of cofactors and vitamins, and glycan biosynthesis and metabolism. Meanwhile, entire males and castrated males were more enriched in KEGG pathways related to membrane transport, cancers: overview and infectious diseases: parasitic function. Given that a previous study showed that the composition of intestinal microflora is related to the immune system status and health of the host [51], the differences we observe may suggest differences in immune function between the sexes.

Compared with entire males and castrated males, female group had functional enrichment in energy production and conversion, coenzyme transport and metabolism, and cell wall/membrane/envelope biogenesis by COG analysis. The entire males showed enrichment in COG pathways related to amino acid transport and metabolism and carbohydrate transport and metabolism. Furthermore, the fecal microbes of entire males and castrated males showed an enrichment over females in some unknown functions. Overall, the function of fecal microorganisms in females was significantly different from that in entire males and castrated males. These differences reveal the differences in the regulation of basic functions of intestinal microorganisms, and could signal differences between sexes in gut function and immune function.

Future studies should look more closely at the relationship between microbiome composition and sex hormones, which would produce a more comprehensive understanding of how sex differences lead to differences in intestinal microbes.

## 5. Conclusions

In general, our research has shown that there are clear differences in the gut microbiomes of different sexes of Hainan special wild boar. The intestinal microbial diversity of female pigs is higher than that of entire male pigs and castrated male pigs, though castration can increase the intestinal microbial diversity of male pigs. The gut microbial communities of entire male pigs and castrated male pigs were significantly different from those of female pigs in function, but there was no significant difference between entire male pigs and castrated male pigs. Therefore, sex should be taken into account in futures studies of intestinal microorganisms in pigs. More specifically, this study provides an important reference for future research on the intestinal microbial diversity of the Hainan special wild boar.

## Supplementary Materials

The followings are available online at https://www.mdpi.com/2076-2615/10/9/1553/s1, Supplementary Figure S1: Sample dilution curve. Abscissa is the number of randomly selected sequencing bars, and ordinate is the number of OTU clustered based on the number of sequencing bars. Each curve represents a group of samples and is marked with different colors. Supplementary Figure S2: Phylogenetic tree of OTU at the level of genus taxonomy. The same color generic name represents the same phylum to which it belongs. Each branch in the evolutionary tree represents a species, and the length of the branch is the evolutionary distance between the two species. Supplementary Figure S3: The histogram of variance analysis between groups at the phylum level. Abscissa represents species and ordinate represents relative abundance of species. The columns of different colors represent the samples of the corresponding groups (* *p* < 0.05, ** *p* < 0.01). Supplementary Figure S4: The histogram of variance analysis between groups at the genus level. The Abscissa represents the species (the top 20 species with the lowest *p*-value were shown), and the ordinate represents the relative abundance of the species. The columns of different colors represent the samples of the corresponding groups (* *p* < 0.05, ** *p* < 0.01). Supplementary Table S1: Composition and Analysis of Diet for fattening Pigs. Supplementary Table S2: Statistics of sample sequencing data processing results. Sample ID is sample name; PE Reads is sequencing double-terminal reads number; Raw Tags is double-terminal reads splicing original sequence number; Clean Tags is original sequence number; Effective Tags is Clean Tags effective sequence number after filtering chimera; AvgLen (bp) is sample average sequence length; GC (%) percentage of bases of G and C types to total bases. Q20 (%) is the percentage of bases with mass value greater than or equal to 20 in total bases; Q30 (%) is the percentage of bases with mass value greater than or equal to 30 in total bases; Effective (%) is the percentage of Effective Tags in PE Reads.
