# Supplementary material for: Sex Differences in Intestinal Microbial Composition and Function of Hainan Special Wild Boar
Source: Animals (Basel). 2020 Sep 2;10(9):1553. doi: 10.3390/ani10091553 (PMC7552319; doi:10.3390/ani10091553)
Supplement: Supplementary file 1 [file animals-10-01553-s001.zip › supplementary Table S1-S2.docx]

| **Items** | **Diet** |
| --- | --- |
| **Ingredients（%）** |  |
| Corn | 27.00 |
| Soybean meal | 16.00 |
| Cassava | 30.00 |
| Wheat bran | 8.00 |
| Rice bran | 15.00 |
| Premix | 4.00 |
| Total | 100.00 |
| **Nutrient levels** | |
| Crude protein (%) | 14.01 |
| Digestible energy (Mcal/kg) | 3.46 |
| Crude fat (%) | 1.84 |
| Crude fiber (%) | 7.45 |
| Crude ash (%) | 8.17 |
| Calcium (%) | 0.52 |
| Available phosphorus (%) | 0.43 |
| NaCl (%) | 0.51 |

**Supplementary Table S1** Composition and Analysis of Diet for fattening Pigs^1^

^1^ Fattening pig feed is produced by Haikou Shuangbaotai Feed Co., Ltd.

**Supplementary Table S2. Statistics of sample sequencing data processing results.**

| Sample ID | PE Reads | Raw Tags | Clean Tags | Effective Tags | AvgLen(bp) | GC(%) | Q20(%) | Q30(%) | Effective(%) |
| --- | --- | --- | --- | --- | --- | --- | --- | --- | --- |
| FE112 | 212807 | 193392 | 179268 | 175445 | 416 | 53.32 | 96.87 | 94.08 | 82.44 |
| FE12 | 300533 | 271377 | 252376 | 248435 | 416 | 53.13 | 96.91 | 94.2 | 82.66 |
| FE122 | 303596 | 275366 | 255936 | 251274 | 416 | 53.37 | 97.03 | 94.4 | 82.77 |
| FE142 | 331616 | 304139 | 284740 | 279508 | 413 | 53.41 | 97.08 | 94.5 | 84.29 |
| FE152 | 321319 | 289520 | 268176 | 263312 | 415 | 53.46 | 96.85 | 94.07 | 81.95 |
| FE22 | 279670 | 248830 | 230246 | 225321 | 414 | 53.61 | 96.74 | 93.89 | 80.57 |
| FE62 | 204353 | 185777 | 173056 | 167782 | 415 | 53.39 | 96.94 | 94.26 | 82.1 |
| FE72 | 222469 | 198315 | 182405 | 178966 | 417 | 53.21 | 96.69 | 93.8 | 80.45 |
| FE82 | 244974 | 218766 | 202641 | 198251 | 415 | 53.24 | 96.91 | 94.21 | 80.93 |
| FE92 | 167948 | 151253 | 140164 | 137639 | 416 | 53.34 | 96.87 | 94.11 | 81.95 |
| EM112 | 258269 | 230812 | 213095 | 207053 | 418 | 53.18 | 96.87 | 94.2 | 80.17 |
| EM12 | 210752 | 191658 | 178668 | 168036 | 415 | 53.42 | 96.9 | 94.22 | 79.73 |
| EM22 | 235355 | 210517 | 193585 | 181544 | 416 | 53.19 | 96.74 | 93.95 | 77.14 |
| EM32 | 217510 | 194287 | 178710 | 166558 | 420 | 52.69 | 96.78 | 94.04 | 76.57 |
| EM42 | 154596 | 139392 | 129109 | 119659 | 416 | 53.99 | 96.79 | 93.95 | 77.4 |
| EM52 | 228434 | 207158 | 190867 | 177183 | 422 | 53.28 | 96.96 | 94.28 | 77.56 |
| EM62 | 258148 | 233245 | 216159 | 205346 | 416 | 53.31 | 96.84 | 94.11 | 79.55 |
| EM72 | 198380 | 177324 | 164582 | 161537 | 414 | 53.29 | 97 | 94.36 | 81.43 |
| EM82 | 157450 | 141948 | 130657 | 126262 | 419 | 53.07 | 97.02 | 94.42 | 80.19 |
| EM92 | 241091 | 219095 | 202939 | 194145 | 417 | 52.88 | 97 | 94.39 | 80.53 |
| CM102 | 149697 | 135548 | 124936 | 116723 | 418 | 54.52 | 96.75 | 93.84 | 77.97 |
| CM112 | 248730 | 225842 | 210724 | 203854 | 413 | 53.6 | 96.81 | 94.09 | 81.96 |
| CM12 | 305076 | 273577 | 253387 | 247382 | 417 | 53.22 | 96.96 | 94.3 | 81.09 |
| CM122 | 282705 | 256997 | 239550 | 224782 | 413 | 53.96 | 97.07 | 94.47 | 79.51 |
| CM132 | 291753 | 267894 | 250685 | 245100 | 414 | 53.17 | 97.04 | 94.45 | 84.01 |
| CM42 | 288802 | 264699 | 247001 | 241878 | 416 | 53.38 | 97.07 | 94.48 | 83.75 |
| CM52 | 201758 | 184102 | 171746 | 163401 | 412 | 53 | 96.92 | 94.27 | 80.99 |
| CM62 | 158759 | 146348 | 136384 | 128658 | 416 | 53.61 | 96.98 | 94.27 | 81.04 |
| CM72 | 137494 | 124540 | 115307 | 108980 | 417 | 53.81 | 96.93 | 94.18 | 79.26 |
| CM92 | 181577 | 163515 | 151162 | 142936 | 416 | 53.84 | 96.79 | 93.97 | 78.72 |

Sample ID is sample name; PE Reads is sequencing double-terminal reads number; Raw Tags is double-terminal reads splicing original sequence number; Clean Tags is original sequence number; Effective Tags is Clean Tags effective sequence number after filtering chimera; AvgLen (bp) is sample average sequence length; GC (%) percentage of bases of G and C types to total bases. Q20 (%) is the percentage of bases with mass value greater than or equal to 20 in total bases; Q30 (%) is the percentage of bases with mass value greater than or equal to 30 in total bases; Effective (%) is the percentage of Effective Tags in PE Reads.
