# Supplementary figures and images for: Sex Differences in Intestinal Microbial Composition and Function of Hainan Special Wild Boar
Source: Animals (Basel). 2020 Sep 2;10(9):1553. doi: 10.3390/ani10091553 (PMC7552319; doi:10.3390/ani10091553)

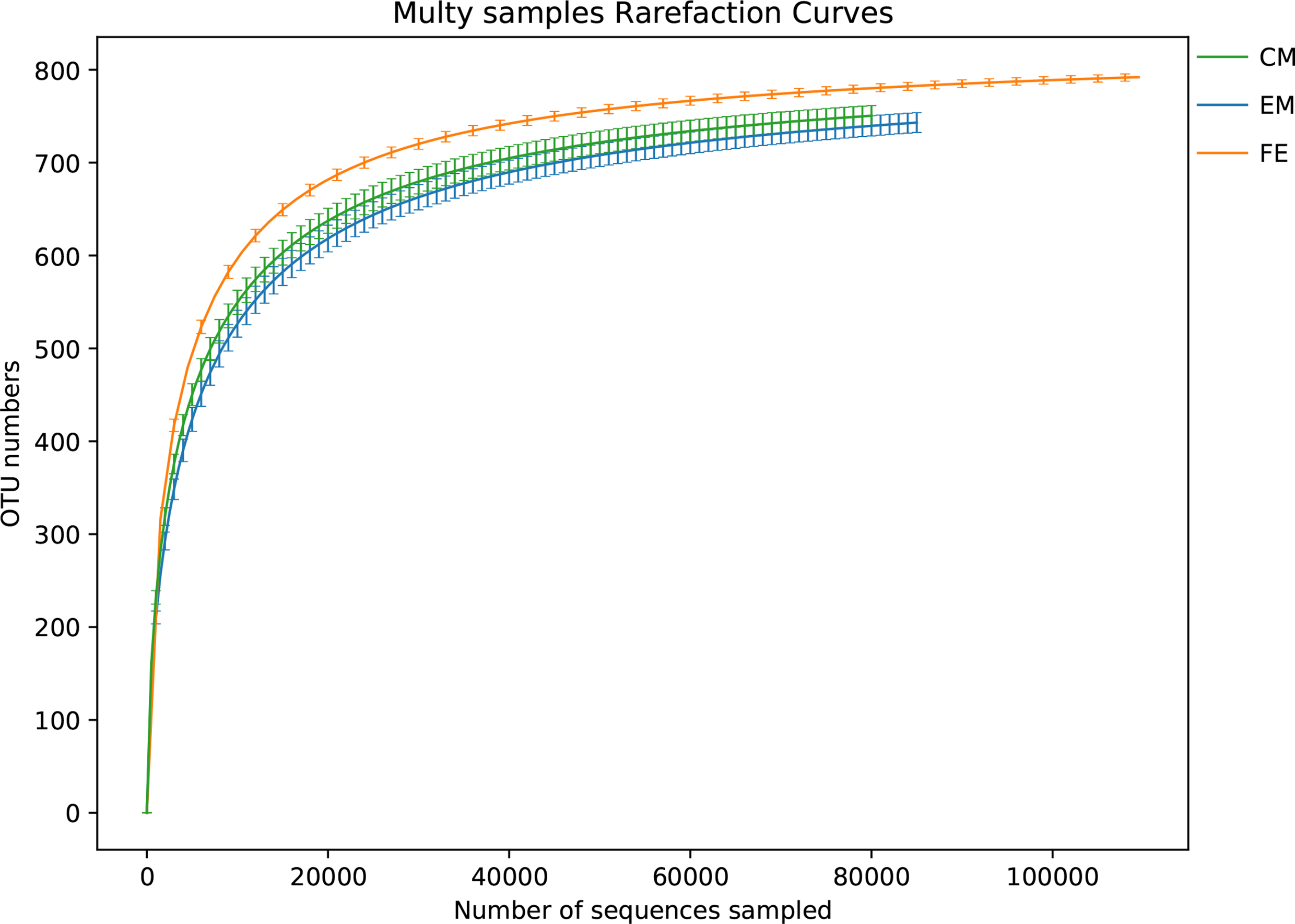

Supplement: Supplementary file 1 [file animals-10-01553-s001.zip › Supplementary Figure S1.tif]

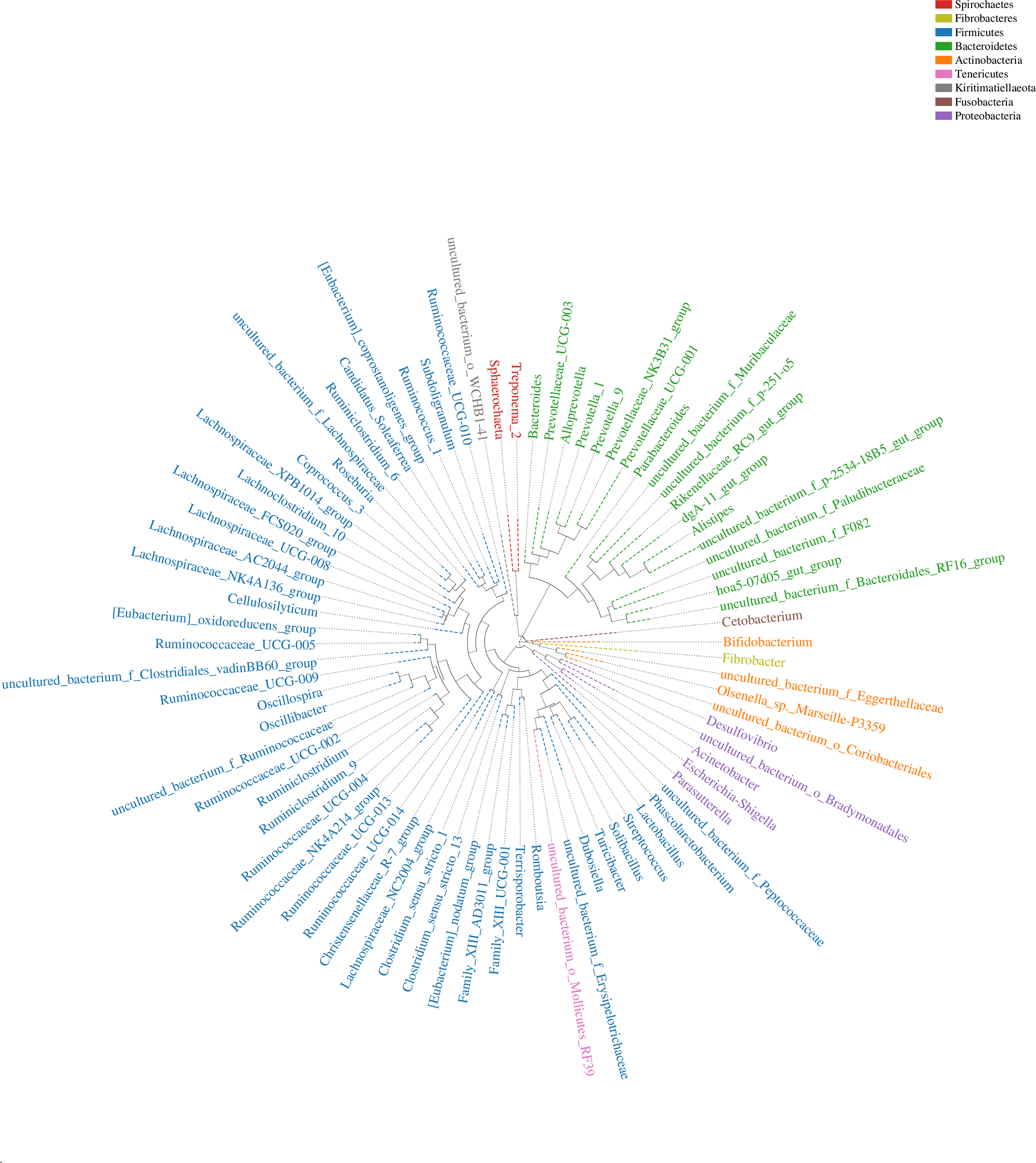

Supplement: Supplementary file 1 [file animals-10-01553-s001.zip › Supplementary Figure S2.tif]

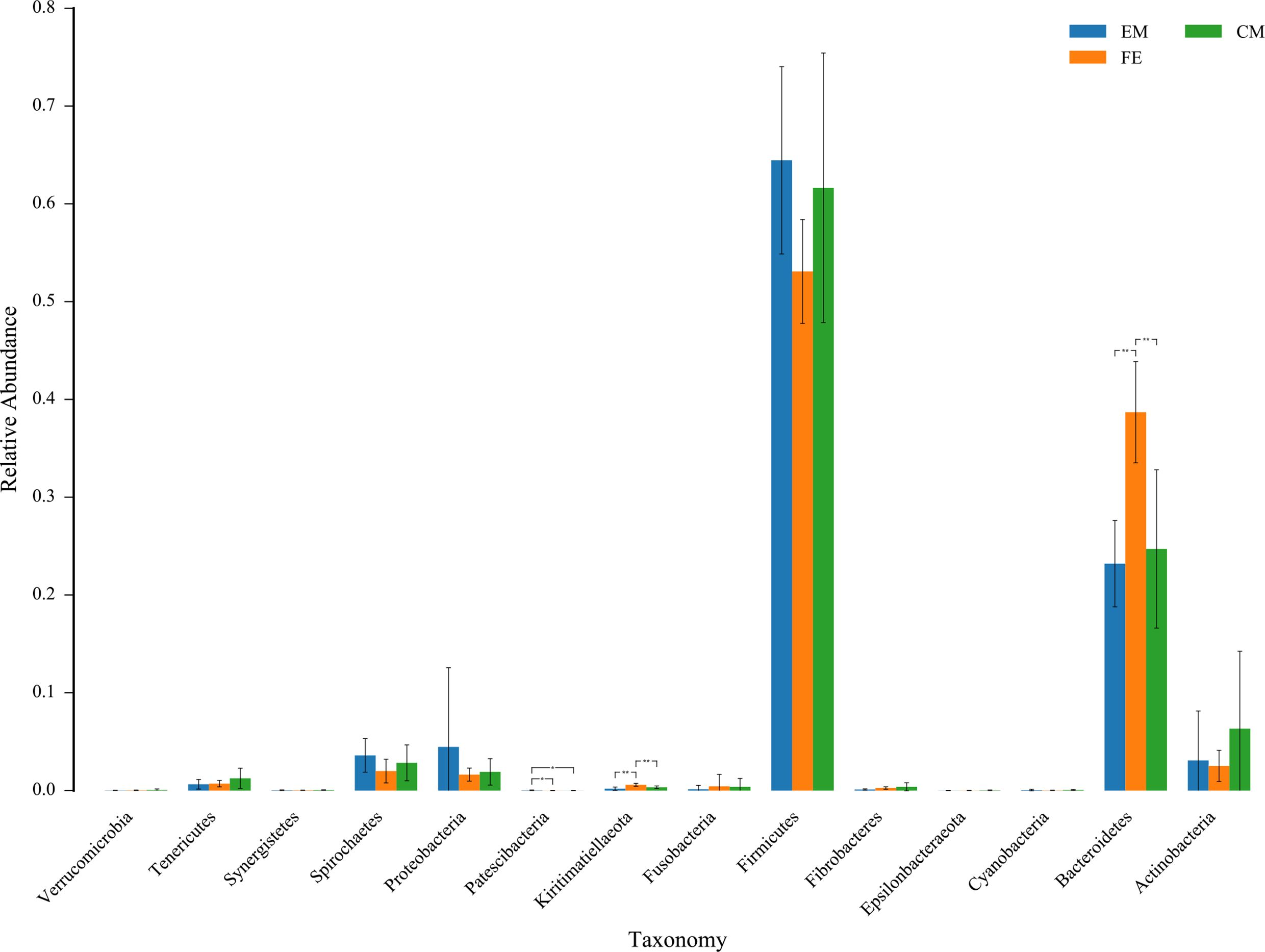

Supplement: Supplementary file 1 [file animals-10-01553-s001.zip › Supplementary Figure S3.tif]

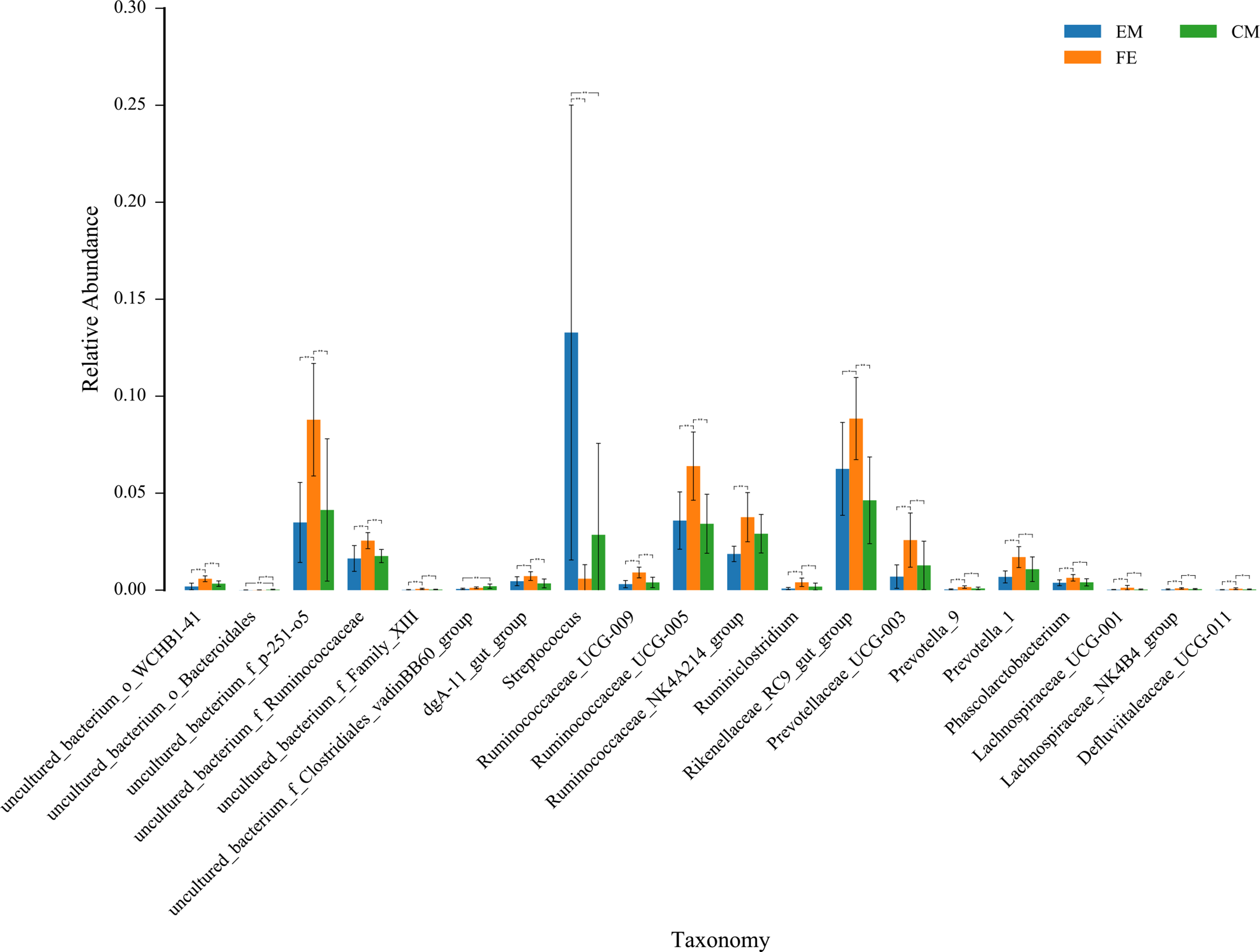

Supplement: Supplementary file 1 [file animals-10-01553-s001.zip › Supplementary Figure S4.tif]
